# Cooperativeness as a Personality Trait and Its Impact on Cooperative Behavior in Young East Asian Adults Who Synchronized in Casual Conversations

**DOI:** 10.3390/bs14110987

**Published:** 2024-10-24

**Authors:** Xiaoqi Deng, Sarinasadat Hosseini, Yoshihiro Miyake, Takayuki Nozawa

**Affiliations:** 1 Department of Computer Science, Tokyo Institute of Technology, Tokyo 152-8550, Japan; deng.x.ac@m.titech.ac.jp (X.D.); hosseini.sarinasadat@gmail.com (S.H.); miyake@c.titech.ac.jp (Y.M.); 2 Department of Intellectual Information Engineering, University of Toyama, Toyama 930-8555, Japan

**Keywords:** cooperation, communication research, social behavior, brain, cognitive science

## Abstract

Cooperation is essential in social life, involving collaborative efforts for mutual benefits. Individual differences in the cooperativeness trait are pivotal in these interactions. A single-group pretest–posttest design was used in this study to determine if Duchenne smiling with gaze and inter-brain synchrony (IBS) during conversation mediates the relationship between cooperativeness and cooperative behavior. The relationships among the variables were examined using mediation analysis and path analysis. We hypothesized that Duchenne smiling with gaze would mediate cooperativeness’ impact on cooperative behavior, while expecting IBS in the left prefrontal region to predict cooperative behavior. The results demonstrated that cooperativeness significantly predicted Duchenne smiling with gaze and cooperative behavior; however, Duchenne smiling with gaze did not mediate the relationship between them. Additionally, IBS during conversation did not predict successive cooperative behavior. These results suggest dispositional factors like cooperativeness may play a more decisive role than momentary expressional cues or neural synchrony in naturalistic unstructured communication in shaping cooperative behavioral outcomes after the communication. The study highlights how personality traits like cooperativeness shape nonverbal communication and social interactions, implying that interventions aimed at developing cooperativeness could lead to more effective collaboration in social settings.

## 1. Introduction

Cooperation is one of the most central aspects of social behavior and has therefore been heavily studied in social sciences. Cooperation refers to the collaborative effort among individuals or groups to achieve shared goals involving mutual benefits [1]. Cooperativeness is a personality trait representing personal preferences for cooperating with others [2], and an individuals’ cooperativeness has been identified as a determinant of their cooperative interactions with strangers. For example, in research on the personality scale, higher cooperativeness scores predicted the individual’s cooperative behaviors [3], and a high willingness to cooperate in economic games positively correlated to their prosocial behavior in other games [4].

The human smile is a prevalent social display that plays a significant role in cooperative interactions. A rich body of studies indicates that smiling is associated with both the sender’s cooperative intent and the receiver’s level of trust. For example, research has shown that smiles are predictive of cooperative decisions in prisoner’s dilemma games [5], and cooperative and altruistic individuals tend to display higher levels of smiling compared to their non-cooperators [6,7]. Studies also reveal that individuals displaying enjoyment smiles are perceived as more trustworthy and are more likely to be cooperated with [8]. Even in trust games, people show more cooperation towards the strangers who are represented by a smiling photograph [9].

Duchenne smiles, characterized by the activation of the orbicularis oculi and zygomatic major muscles, resulting in raised corners of the mouth and wrinkles around the eyes, are widely recognized as genuine expressions of positive emotion and are closely linked to cooperation. According to Reed et al., individuals displaying Duchenne smiles are more likely to cooperate compared to those with non-Duchenne smiles [5]. Mehu et al. emphasize the role of Duchenne smiles in maintaining cooperative relationships [6]. Moreover, individuals displaying Duchenne smiles are perceived as more generous, and Duchenne smiles could indicate sociability and altruism [10]. Despite the ease of faking smiles [11], Duchenne smiles are regarded as reliable indicators of cooperative intent. The involvement of both emotional-driven orbicularis oculi and zygomatic major muscles makes Duchenne smiles difficult to falsify voluntarily [12]. Research consistently demonstrates a correlation between Duchenne smiling and cooperativeness. For instance, Brown et al. observed greater activity in the orbicularis oculi muscles among altruists [7], suggesting that Duchenne smiles may authentically reflect cooperative tendencies, and this effect is particularly pronounced compared to non-Duchenne smiles [5]. The relationship between Duchenne smiles and cooperation has been studied in various cultural contexts. Most of the studies have focused on Western cultures [5,6,7,10,13,14,15], while research in Eastern cultures remains limited. Notably, Schug et al. [16] found that cooperators exhibited more emotional expressions, including the Duchenne smile, among participants from a major research university in Japan. In this study, we investigated the connection between the Duchenne smile and cooperation within an Eastern cultural context, aiming to contribute to the understanding of this relationship across diverse cultures.

Humans possess particularly visible eyes compared to other primates. This adaptation is believed to have evolutionary advantages, facilitating complex social behaviors such as cooperation, the communication of emotions, and the coordination of group activities [17]. Tomasello et al. propose a theory suggesting that human-like eyes evolved due to selective pressures favoring improved cooperative and communicative abilities, essential for mutualistic social interactions, including joint attention and visually based communication like pointing. Their research demonstrated that humans especially rely on eyes during gaze-following scenarios, suggesting that eyes evolved a novel social function in human evolution, primarily to facilitate cooperative social interactions [17]. Other research also illustrates that gaze direction serves as a pivotal cue in human social interactions, conveying valuable information about attention, interests, and intentions [18]. Gaze direction indicates where an individual’s focus lies and can effectively redirect an observer’s attention [19]. Moreover, gaze direction significantly influences the perception of emotional facial expressions; when aligned with the underlying behavioral intent, it enhances the interpretation of those expressions. In the context of smiling, a direct gaze concurrent with a smile amplifies how the smile is perceived by others [20]. This combination not only guides attention and focus but also facilitates the accurate interpretation of facial expressions, potentially making smiles more effective in signaling cooperative intent [21].

Despite existing research suggesting that Duchenne smiles may refer to an inner state of cooperativeness and signal cooperative intent [6,7], the specific influence of gaze direction during Duchenne smiling on cooperation remains unexplored. In the current study, a hypothesis has been proposed to explain the relationships between Duchenne smiling with a direct gaze, cooperativeness, and cooperative behavior. We examined gaze direction during Duchenne smiling across the course of a five-minute conversation among pairs of same-sex strangers. We were interested in the relationship between synchronized smiling across the conversation with gaze or gaze aversion and the cooperation results in the prisoner’s dilemma game before and after the conversation, which measured the individuals’ cooperativeness and their cooperative behavior. Based on previous research, we expected that the index of the individuals’ Duchenne smiling with gaze would (1) be predicted by their cooperativeness and (2) predict their cooperative behavior. We also examined the mediative effect of the Duchenne smile with gaze on the relationship between cooperativeness and cooperative behavior.

In considering the underlying neural mechanism of communication, we also took into account interpersonal neural synchrony. Recently, there has been growing interest in studying interpersonal neural synchrony using hyperscanning techniques in experimental setups that replicate real-world situations within cognitive neuroscience [22,23,24]. Hyperscanning is a form of experiment in which the brain activities of two or more participants are recorded simultaneously when they are interacting [25]. Interpersonal neural synchrony (i.e., inter-brain synchrony, IBS) refers to the similarity between two neural signals coming from different brains [26]. A considerable amount of studies have postulated inter-brain synchronization as well as intra-brain synchronization as mechanisms underlying communication [27]. IBS during social interactions has been linked to prosocial behaviors such as cooperation, coordination, and collective performance [28,29,30]. Significant IBS was observed in the dorsolateral prefrontal area of the dyads, showing higher subjective cooperativeness during joint-drawing tasks [31]. Specifically, a rich body of research has reported that the IBS observed in the frontal regions (including the left inferior frontal cortex and frontal pole) was correlated with prosocial interactions. For instance, parents’ and children’s brain activities that synchronized in the dorsolateral prefrontal and the frontopolar cortex predicted their cooperative performance [32], and the synchrony of the anterior cingulate cortex and the prefrontal areas between the brains of paired subjects was observed while they were playing the prisoner’s dilemma game [33].

Recent evidence highlights the correlation between IBS and personality traits, underscoring the pivotal role of personality in understanding synchronized interpersonal neural activities [34,35]. Recently, two neuroimaging studies using functional magnetic resonance imaging (fMRI) and electroencephalography (EEG) provided evidence linking IBS when viewing naturalistic stimuli to personality profiles [34]. From a neural perspective, studies have also explored how the trait of cooperativeness influences structural connectivity. For instance, in a study investigating the associations between cooperativeness and fiber connectivities from the striatum to nine subcortical and cortical regions, cooperativeness had been reported to be related to the connectivity between the caudate and anterior cingulate cortex and the ventrolateral prefrontal cortex [36]. However, the existing literature on the relationships between IBS, personality traits, and cooperative behavior remains limited, especially concerning the use of functional near-infrared spectroscopy (fNIRS). By incorporating fNIRS into this research, we aim to provide new insights into the neural correlates of cooperation, contributing to a more comprehensive understanding of how brain function relates to cooperative outcomes.

In the current study, we examined the effect of IBS on the left prefrontal region, which is reported to be associated with smiling [12,37], as well as involved in prosocial interactions. We focused on the IBS during an unstructured conversation with spontaneous emergent behaviors. Dyads were recruited to have a 10 min conversation during which their facial expressions and brain activities were recorded. Before and after the conversation, the dyads were asked to perform the prisoner’s dilemma game to measure their cooperativeness and cooperative behavior. The brain activities of all the dyads were recorded using fNIRS. We expected to observe IBS during the conversation. We also expected that cooperativeness would predict IBS during the conversation, which would in turn predict successive cooperative behavior.

**Figure 1:**
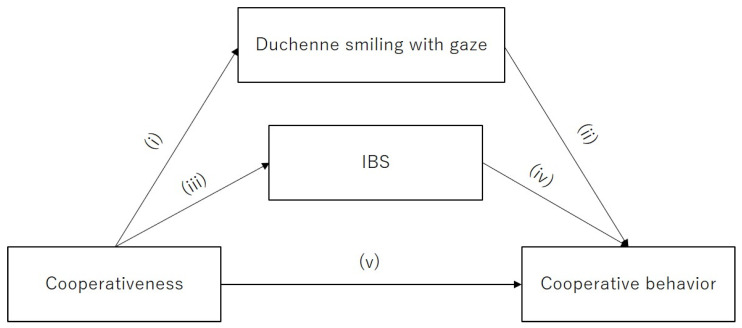
Hypothetical model. The hypotheses are demonstrated as the following: (i) cooperativeness predicts Duchenne smiling with gaze; (ii) Duchenne smiling with gaze predicts cooperative behavior; (iii) cooperativeness predicts IBS; (iv) IBS predicts cooperative behavior; and (v) cooperativeness predicts cooperative behavior.

In considering the effect of cooperativeness and Duchenne smiling with gaze on cooperative behavior, we performed a path analysis to test the hypotheses, as shown in Figure 1. Based on the hypothesis, we expected that (i) cooperativeness would predict Duchenne smiling with gaze; (ii) Duchenne smiling with gaze would predict cooperative behavior; (iii) cooperativeness would predict IBS; (iv) IBS would predict cooperative behavior; and (v) cooperativeness would predict cooperative behavior.

## 2. Materials and Methods

### 2.1. Participants

90 university and college students in Tokyo, Japan were recruited for this study. The participants in this study were healthy students aged between 20 and 30 years, all of whom had normal or corrected-to-normal vision and identified as right-handed. A convenience sampling method, a type of non-probability sampling method, was used in this study. By using this sampling method, we focused on recruiting participants from an accessible group of university students. To ensure a relevant and unbiased sample, none of the participants were majoring in psychology or other related fields. This selection criterion was established to minimize the influence of prior knowledge in psychology on the study’s outcomes. This approach allowed us to minimize bias while ensuring an adequate sample size. According to Kline [38], an adequate sample size should always be 10 times the amount of the parameters in a path analysis, and the best sample size should be 20 times the number of parameters in a path analysis. Iacobucci [39] suggested that a sample size of at least 50 is acceptable. According to Sim et al. [40], mediation analysis using the bootstrapping method with a sample size range of 50 to 130 is acceptable. Therefore, we reached the current number of participants. We also conducted a post hoc power analysis for the path analysis, and the power was 0.63. For the mediation analysis, the power was 0.81.

The participants received JPY 2000 (approximately USD 17.5) at participation and were able to receive another payment of up to JPY 450 (approximately USD 3.9) based on the outcome of the prisoner’s dilemma (see below). The participants were randomly assigned to 45 same-sex and same-nationality dyads (21 female participants and 24 male participants), without any knowledge of their assigned partners prior to the experiment.

The participants ranged in age from 20 to 30 years, with an average age of 22 years old. 68.9% of the participants were Japanese and 31.1% of the participants were Chinese. This study was approved by the Ethics Committee of the Tokyo Institute of Technology. Written informed consent was provided by all the participants in this study. The participants in this study were the same as those in our previous study [41], and thus the descriptions in the following two subsections are mostly reproduced from that paper.

### 2.2. Experimental Design

A single-group pretest–posttest design was used in this study to determine if Duchenne smiling with gaze and inter-brain synchrony (IBS) during conversation mediate the effect of cooperativeness on cooperative behavior. This research design was selected to explore potential causal relationships between an independent variable and a dependent variable, with a focus on the role of the mediator. Such a design is common in research where treatment and outcomes are respectively measured before and after the manipulation of the group.

### 2.3. Instruments

Two Sony FDR-AX45 (Tokyo, Japan, 4 K, 24 Hz) digital camcorders were used to capture the facial expressions of the participants. Two wearable two-channel continuous fNIRS instruments (HOT-1000, Hitachi High-Technologies Corporation, Tokyo, Japan) were used to continuously record the brain activities of the participants.

### 2.4. Data Collection Procedure

The data collection procedure contained three data collection sessions: game session 1, a conversation session, and game session 2. The three sessions were designed to collect data for measuring cooperativeness (game session 1), Duchenne smiling with gaze and IBS (the conversation session), and cooperative behavior (game session 2), respectively.

Before the experiment, the participants were instructed to sit on opposite ends of a meeting table with 1 m of distance in between them and with a divider to separate them. This was to ensure that they would not see each other or have any communication before the conversation session started. The participants were then given a description of the study and the procedures to be undertaken in the study. The introduction did not inform the participants of the real objectives of the research or the real purpose of the experiment, therefore leading the participants to believe that they were participating in research about personality and communication. This was carried out to avoid the participants’ conscious attention to their partners’ smiles and cooperation. Following the introduction, the participants were given a consent form to review and sign.

#### 2.4.1. Game Session 1

The participants were first told that they would participate in a one-shot prisoner’s dilemma game, and were made to believe that they would play the game with an unseen experimenter who they would not have any interaction with thereafter. This was carried out to measure their cooperativeness with strangers via the result of the game. The details of the prisoner’s dilemma game and how the results of the game were treated to measure cooperativeness are described in Section 2.5. The participants were assured that the other participants would not know their game decisions and they were not informed about their own profits from the game until the experiment was finished.

#### 2.4.2. Conversation Session

After the one-shot prisoner’s dilemma game, the participants were instructed to assume a relaxed posture in the seat for a five-minute rest, during which two sets of wearable two-channel fNIRS instruments were placed on their heads. Two Sony FDR-AX45 digital camcorders were placed approximately 0.5 m behind of each participant and were used to record the facial behavior of the participant on the opposite end of the table. The participants were aware that in addition to the fNIRS brain measurement, they were being videotaped. But, the participants were not informed that their expressions would be coded for further analysis, in order to avoid their conscious attention to their own smiles. Following the test session, the divider was removed for the participants to take part in a ten-minute, face-to-face, unstructured “getting to know you” conversation, during which they were told that they could talk about any topic. The participant’s brain activities were recorded by the two-channel fNIRS instruments and the participants’ facial behaviors during the conversation session were video-recorded for the full 10 min at 30 frames per second with the participants’ knowledge and consent. The recorded facial expressions were further coded by two certified coders via replaying the video frame by frame. The details of how the facial expressions were coded and how the coded facial expressions were calculated as the index of Duchenne smiling with gaze is described in Section 2.6. The signals of the recorded brain activities were then delivered to a computer to calculate the index for measuring IBS. The detailed process of artifact reduction and the calculation of the signal are described in Section 2.7 and Section 2.8.

#### 2.4.3. Game Session 2

Immediately following the conversation, the participants were once again divided by the divider and instructed to take part in a one-shot prisoner’s dilemma game with their conversation partners. This was carried out to measure their cooperative behavior with their conversation partner via the result of the game. The details of the prisoner’s dilemma game and how the results of the game were treated to measure the participants’ cooperative behavior are described in Section 2.5. After completing the experiment, the participants were informed of the real purpose of the experiment and the plan for facial expression usage in the analysis. They were then given a complete description of the real aims of the research and the actual procedure of the experiment, and signed a consent form to confirm that they still agreed to participate in the research and to authorize the use of their personal data and video records for scientific purposes.

**Figure 2:**
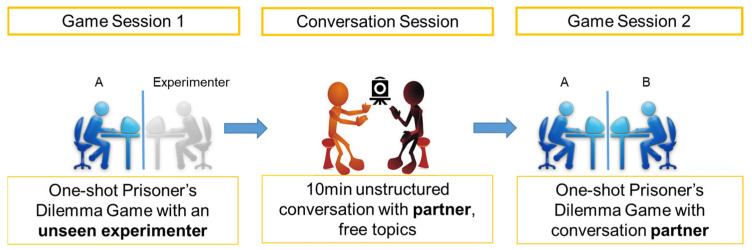
Experiment procedure demonstrating each session of the experiment in a step-by-step style.

The experiment procedure is shown in Figure 2.

### 2.5. Prisoner’s Dilemma Game

The prisoner’s dilemma game was conducted using an exchange protocol [42]. Following this protocol, the participants could choose different levels of cooperation rather than choose between cooperating or defecting. In the game, each participant was provided with an endowment of JPY 150 (approximately USD 1.31) and was asked to decide how much of the endowment to give to their game partner. The provided money was then doubled and given to their partner. The participant retained the money that they did not give away. If both of the participants provided JPY 150 (fully cooperated), each received JPY 300. If one participant fully cooperated and provided JPY 150, and the other participant offered no money, the one who fully cooperated earned nothing, and the one who completely defected earned JPY 450. If both of the participants chose to give away nothing, each earned JPY 150 (mutual defection). The participants earned more by giving away less regardless of their partner’s offer level. Therefore, these outcomes corresponded to the four cells in the standard prisoner’s dilemma matrix. The outcome possibilities were clearly outlined for the participants in a chart, as shown in Table 1.

The prisoner’s dilemma game was conducted twice: first, it was conducted with an assumed stranger (an experimenter who they would not interact with or know their information), and then with their partner after the conversation session. The proportion of the sum of the money that the pair offered before the conversation was used as a measure of the pair’s cooperativeness. The proportion of the sum of the money that the pair offered after the conversation was used as a measure of the pair’s cooperative behavior.

### 2.6. Behavior Analysis

Analyses of the participants’ facial behaviors during the conversations were only conducted for 5 min directly before the prisoner’s dilemma game. By focusing the analyses on the 5 min of the clip that occurred directly before the prisoner’s dilemma game, we aimed to capture the facial actions that were highly relevant to cooperative behavior.

#### 2.6.1. FACS Coding

Ekman and Friesen’s Facial Action Coding System (FACS) was used to measure facial behavior [43]. Smiles were coded as either present or absent in 1 s intervals for the 5 min clip. Each second, if a smile was present, it was coded as either a Duchenne smile (AU_s_ 6 + 12, lip corners raising up as well as the presence of cheek movement and “crow’s feet” wrinkles, indicating the contraction of the orbicularis oculi muscles) or a non-Duchenne smile (AU 12, rise of the lip corners), following the use of the FACS. If a Duchenne smile was present during a second, that smile was coded as 1 for that second.

All the videos of the 5 min clip were coded by a certified coder. Approximately 20 percent of the overlapping videos were coded by another certified coder, in order to assess their reliability. The average pairwise reliability across the coders, based on the intraclass correlation coefficient (ICC), was 0.917 using random effects.

#### 2.6.2. Gaze Direction Coding

The individual participants’ gaze direction was coded as a gaze (if looking at their partner) or gaze aversion (if not looking at their partner) in 1 s intervals for the 5 min clip by a coder. Each second, the gaze direction was given a score of 1 (gaze) or 0 (gaze aversion), thus generating a series of binary data. Approximately 10 percent of the overlapping videos were coded by another coder to assess their reliability. The average pairwise reliability across the coders, based on the intraclass correlation coefficient (ICC) was 0.759 using random effects.

Duchenne smiling with gaze was coded as 1 when both the Duchenne smile code and the gaze direction code were 1. The proportion of time Duchenne smiling with gaze occurred was calculated by counting the total number of seconds it was observed and dividing this count by the total observation time of 300 s. The index was calculated as the dyadic average proportion of time that Duchenne smiling with gaze occurred. The index was saved to use in the following statistical analysis.

### 2.7. fNIRS Measurement

A variety of neural scanning techniques have been used to simultaneously record brain activities such as functional near-infrared spectroscopy (fNIRS), electroencephalography (EEG), and functional magnetic resonance imaging (fMRI). A review of the current methods used in hemodynamic and electrophysiological hyperscanning studies showed that fNIRS and Wavelet coherence were the most common neuroimaging modalities and methods [25]. In the current study, we employed the fNIRS hyperscanning approach to acquire brain activities from all the dyads, whose prefrontal neural activities were acquired with wearable two-channel continuous fNIRS instruments (HOT-1000, Hitachi High-Technologies Corporation, Tokyo, Japan). The fNIRS probe comprised one infrared light source (wavelength 810 nm) and two light detectors at a distance of 1.0 and 3.0 cm from the light source, respectively. Neural activity data were collected through the four detectors of the two probes. The sum of the concentration of the oxyhemoglobin and deoxyhemoglobin changes, defined as the concentration of the total-hemoglobin (total-Hb) changes, on the optical path of the source-detector were calculated from the changes in the detected light intensities using the modified Beer–Lambert law [44,45]. NIRS probes were positioned per the international 10–20 electrode system used in electroencephalography (EEG), such that the center of Fp1 and Fp2 matched the center of the optode component in the right probe which covered the rostral limit of superior frontal gyrus, and the left probe covered the left prefrontal lobe [27,46].

The participants were instructed to maintain a relaxed posture while sitting and to limit sudden movements of their heads as much as possible. The neural signals acquired by the fNIRS of the 36 pairs of participants were used to analyze IBS. Data from one of the pairs of participants were excluded due to recording failure during the conversation session. Eight pairs of participants were excluded due to failing to attach the probe to cover their left prefrontal region of interest.

### 2.8. Artifact Reduction Methods for fNIRS Data

The fNIRS signals were preprocessed using R. First, the drift components were removed from each signal using linear detrending. Then, dual source-detector regression [47] was applied to regress out the shallow-tissue signal component (dominated by non-neuronal systemic and motion-related noises) captured by the 1.0 cm source-detector channel from the deep-tissue signal component (contains both the non-neuronal shallow and neural deep components) captured by the 3.0 cm source-detector signal to extract the neural component. The dual source-detector regression method is expressed in the following formula:

$$x_{deep}=a_{0}+a_{1}x_{shallow}+x_{neural}$$

### 2.9. Analysis of IBS

The left prefrontal inter-brain synchrony for each pair of participants was calculated using MATLAB (version R2023b; MathWorks Inc., Natick, MA, USA). With the neural signals extracted through the preprocessing steps above, wavelet transform coherence (WTC) [48,49] was computed using the cross wavelet and wavelet coherence toolbox (http://grinsted.github.io/wavelet-coherence/ (accessed on 14 December 2023)). WTC has been widely used in fNIRS hyperscanning research to evaluate a localized correlation coefficient in time–frequency space to capture IBS [22]. According to previous studies [50,51], a larger coherence value should be observed when the participants are interacting. Based on the same rationale, we compared the averaged coherence of the genuine dyads during the last 5 min of the conversation directly before the prisoner’s dilemma game with that of the permutated dyads using multiple two-sample *t*-tests for each period. In order to adjust the *p*-values derived from the multiple statistical tests to correct for the occurrence of false positives, a false discovery rate (FDR) adjustment was applied [52]. Permutated pairs were formed by the participants who were in different pairs. Because the genuine dyads interacted during the conversation, but the permutated dyads did not, a larger averaged coherence should have been observed in the periods sensitive to the interaction. The range of the timescales (Fourier periods) with significantly higher coherence in the genuine dyads compared to the permutated dyads were identified and used as the periods of interest (POIs).

### 2.10. Data Analysis

In this study, we were interested in examining the relationship between cooperativeness, Duchenne smiling with gaze, IBS, and cooperative behavior, with a specific focus on the mediation effect of Duchenne smiling with gaze and IBS on cooperative behavior. We hypothesized that (i) cooperativeness would predict Duchenne smiling with gaze; (ii) Duchenne smile with gaze would predict cooperative behavior; (iii) cooperativeness would predict IBS; (iv) IBS would predict cooperative behavior; and (v) cooperativeness would predict cooperative behavior.

In order to test the hypothesis, we conducted a mediation analysis and a path analysis using R software (version 4.3.2, https://cran.r-project.org/ (accessed on 31 October 2023)).

#### 2.10.1. Mediation Analysis

**Figure 3:**
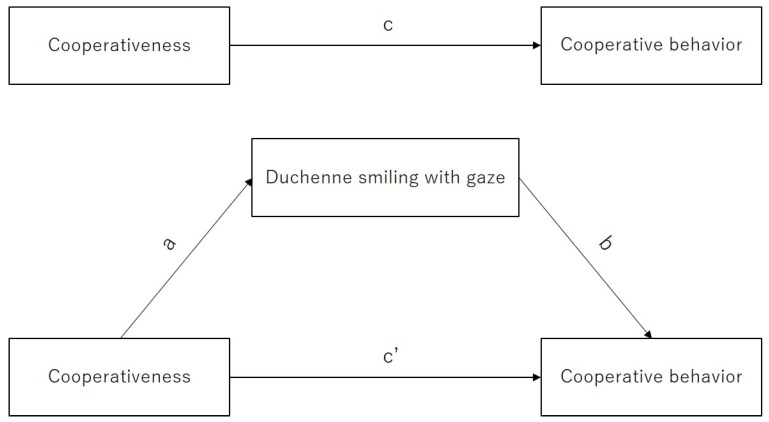
Diagram of hypothesized mediation model. Path (c) determined the total effect of cooperativeness on cooperative behavior; path (a) and (b) determined the indirect effect of cooperativeness on cooperative behavior through Duchenne smiling with gaze; and path (c′) determined the direct effect of cooperativeness on cooperative behavior.

A mediation analysis was conducted following [41,53] via the bootstrapping method to determine if the effect of the independent variable (cooperativeness) on the dependent variable (cooperative behavior) could be explained by the mediating variable (Duchenne smiling with gaze). The bootstrap re-samples were set to 1000 to ensure replicates. A pathway (Figure 3) was specified a priori, showing the mediation model in which path c determined the total effect of cooperativeness (independent variable) on cooperative behavior with no consideration of mediator variables; path a and b determined the indirect effect of cooperativeness on cooperative behavior through Duchenne smiling with gaze; and path c’ determined the direct effect of cooperativeness on cooperative behavior after removing the contribution of Duchenne smiling with gaze. The mediation analysis was conducted using the mediation package in R software, which computes the total effect of the independent variable on the outcome, the Average Causal Mediation Effects (ACME) for the indirect effect, and the Average Direct Effects (ADE) for the direct effect. A mediator was considered to have a mediational effect when (1) the indirect effect (i.e., path a × path b) of cooperativeness on cooperative behavior via smiling with gaze was significant; and (2) the bias-corrected 95% CI around the indirect effect from 1000 bootstrap re-samples excluded zero.

#### 2.10.2. Path Analysis

To analyze the role of IBS during conversations in successive cooperative behavior, a path analysis was conducted. Path analysis is a type of structural equation modeling that examines relationships among a set of observed variables, which allows the study of multiple direct and indirect relationships between variables simultaneously [54]. We used path analysis to examine the direct and indirect effect (the mediating effect of IBS) of cooperativeness on cooperative behavior. The path analysis was conducted using a Lavaan package in R software. To assess the model’s goodness of fit, the Normed Chi-Square (χ^2^), the Standardized Root Mean Square Residual (SRMR), and the Comparative Fit Index (CFI) were consulted [55]. A nonsignificant χ^2^, SRMR value of ≤0.08, and a CFI ≥ 0.95 were considered a good fit.

## 3. Results

### 3.1. Linear Regression Results Indicate That Cooperativeness Predicts Duchenne Smiling with Gaze, Which Correlates to Cooperative Behavior

We first conducted linear regression analyses to examine if Duchenne smiling with gaze significantly predicts cooperative behavior. In accordance with our hypothesis, the results showed a significant positive correlation between dyadic average Duchenne smiling with gaze and their cooperative behavior (t(43) = 2.70, *p* = 0.009, *r*^2^ = 0.12). We then tested if Duchenne smiling with gaze is significantly predicted by cooperativeness. The results of linear regression showed that, as hypothesized, dyadic average Duchenne smiling with gaze is significantly predicted by cooperativeness (t(43) = 2.93, *p* = 0.005, *r*^2^ = 0.14).

### 3.2. Mediation Analysis Result Showed No Mediating Effect of Duchenne Smiling with Gaze

The results from the mediation analyses are presented in Table 2. The results indicated a significant total effect of the association between cooperativeness and cooperative behavior (total effect = 0.661, *p* < 0.001). The direct effect between cooperativeness and cooperative behavior was significant (ADE = 0.599, *p* < 0.001); however, the indirect effect of cooperativeness on cooperative behavior via smiling with gaze was not significant (ACME = 0.062, 95% CI: LLCI = −0.056 to ULCI = 0.2, *p* = 0.25). These results suggest that Duchenne smiling with gaze would not be considered a mediator for the relationship between cooperativeness and cooperative behavior.

### 3.3. Two-Sample t-Test Result Showed Enhanced IBS During Conversation

**Figure 4:**
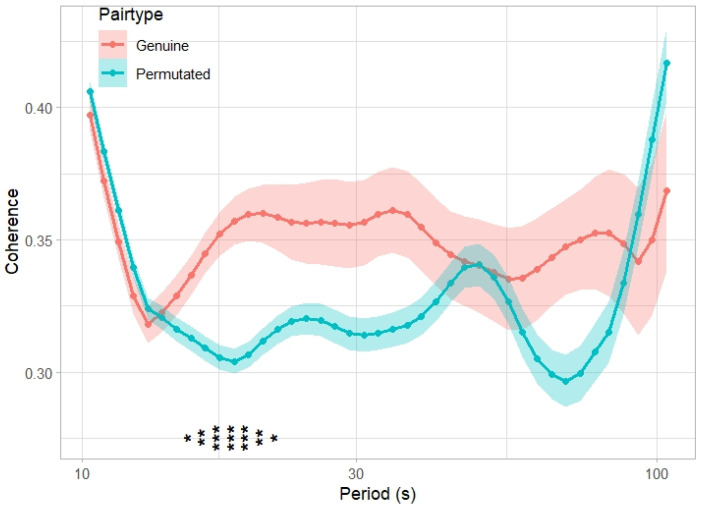
Comparison of the IBS of the left frontal cortex signals from genuine dyads and permutated dyads. Solid lines show the mean of time-averaged WTC over all the genuine and permutated dyads of participants for each pair. Shaded areas show the standard error of the mean (SEM) over the genuine and permutated dyads. Asterisks at the bottom (vertically oriented in the figure) indicate periods with significant differences; *: q < 0.05; **: q < 0.01, ***: q < 0.001 (FDR-adjusted).

To analyze the IBS of the left frontal signals, we compared the WTC from genuine dyads and permutated dyads. First, we generated 180 permutated dyadic WTC data by computing the preprocessed neural signals of randomly paired participants who were not in the same dyad for interacting during the conversations. Then, we performed multiple two-sample *t*-tests to compare the time-averaged coherence value for each timescale between the genuine dyadic data and the permutated dyadic data. False discovery rate (FDR) adjustment [52] was applied to adjust the *p*-values derived from the multiple statistical tests to correct for the occurrence of false positives. The comparison of the IBS of the left frontal cortex signals from the genuine dyads and permutated dyads is shown in Figure 4. Each period with a significantly larger averaged coherence of the genuine dyads was identified. The coherence values in these periods were sensitive to the interaction. We observed that the average coherence values of the genuine dyads between 15.48 and 21.89  s (0.04–0.06  Hz) were significantly higher (FDR-adjusted q < 0.05) than that of the permutated dyads (Table 3), thus these timescales were used as POI for further calculating the index of IBS during the communication. This frequency band was a range that excluded the high- and low-frequency noises that would lead to artificial coherence. The average of the time-averaged coherence values in the identified POI was calculated and used as an index of IBS during the conversation task for the following path analysis.

### 3.4. Path Analysis Result Showed No Mediating Effect of IBS on Cooperative Behavior

**Figure 5:**
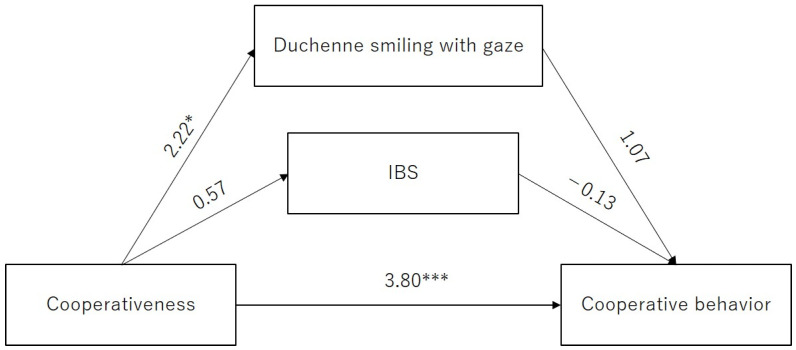
Path diagram of pathways. Cooperativeness positively predicted cooperative behavior (β = 0.64, z-value = 3.80, *p* < 0.001, *r*^2^ = 0.38), and Duchenne smiling with gaze (β = 0.07, z-value = 2.22, *p* = 0.02, *r*^2^ = 0.12). Asterisks indicate significant effects; *: *p* < 0.05; ***: *p* < 0.001.

The results of path analysis are presented in Figure 5. The model demonstrated an acceptable fit (*p* = 0.51, CFI = 1, SRMR = 0.03) and accounted for 38% of the cooperative behavior variance. The z-values in Figure 4 clearly showed that cooperative behavior was predicted positively by cooperativeness (β = 0.64, z-value = 3.80, *p* < 0.001, *r*^2^ = 0.38). However, both the path from Duchenne smiling with gaze (β = 0.83, z-value = 1.07, *p* = 0.28, *r*^2^ = 0.12) and the path from IBS during conversation (β = −0.13, z-value = −0.13, *p* = 0.89, *r*^2^ = 0.01) to cooperative behavior were not significant. In addition, it showed that cooperativeness positively predicted the proportion of time Duchenne smiling with gaze occurred (β = 0.07, z-value = 2.22, *p* = 0.02, *r*^2^ = 0.12).

## 4. Discussion

In the current study, we explored the relationships among cooperativeness, Duchenne smiling with gaze, and IBS during conversation, and their impacts on cooperative behavior. We conducted a mediation analysis to examine the effect of Duchenne smiling with gaze on the relationship between cooperativeness and cooperative behavior. The results showed that cooperativeness significantly predicted Duchenne smiling with gaze and cooperative behavior; however, Duchenne smiling with gaze did not mediate the relationship between them. We further conducted a path analysis to examine the role of IBS during conversation in successive cooperative behavior. The path analysis results showed that cooperativeness directly affected cooperative behavior. Cooperativeness significantly predicted Duchenne smiling with gaze; however, neither Duchenne smiling with gaze nor IBS during conversation predicted successive cooperative behavior.

Our findings indicated several significant associations. Firstly, consistent with our prior study [41], we found that cooperativeness was a robust predictor of both Duchenne smiling with gaze and cooperative behavior. Indeed, the idea that personality plays a central role in cooperation was already expressed decades ago. For example, Chatman and Barsade examined personal cooperativeness as a single-dimension personality characteristic varying from high personal cooperativeness to low personal cooperativeness (individualism) and found that interacting with others is more closely related to one’s personality (those with higher dispositions to cooperate interact more with others) than to the demands of the situation [56]. A recent study in children revealed that cooperativeness predicted more optimal mother–adolescent interaction [57]. However, the majority of recent research emphasizes the role of behavioral mechanisms underlying cooperation from the perspective of animal signals [58]. A rich body of research suggests that smiles are the behavioral mechanism underlying cooperation by signaling cooperative intent. Mehu et al. suggests that the Duchenne smile could be an important signal in the maintenance of cooperative relationships [6]. Reed et al. found that senders expressing smiles would be more likely to cooperate, and this effect was particularly strong for Duchenne smiles compared to non-Duchenne smiles [5]. However, previous studies have not differentiated between cooperativeness and cooperative behavior by assessing participants’ cooperativeness as a personality trait prior to their decision to cooperate. For instance, according to the perspective of reciprocal altruism, cooperation in the prisoner’s dilemma game might indicate an optimistic expectation regarding the perceived partner’s likelihood to cooperate. Similarly, cooperation could stem from a strong predisposition toward cooperating, serving as an expression of individuals’ personality trait of cooperativeness. Consequently, it remains ambiguous whether the observed association between cooperative behavior and smiles reflects the detection of cooperative partners or if both cooperative behavior and smiles are merely influenced by a shared trait determinant, specifically the trait of cooperativeness. Our findings that cooperativeness robustly predicts both Duchenne smiling with gaze and cooperative behavior underscore the role of personality traits in shaping nonverbal communication and subsequent social interactions. However, contrary to our hypothesis and previous literature suggesting the potential mediating role of Duchenne smiling with gaze, our mediation analysis did not support Duchenne smiling with gaze as a mediator in the relationship between cooperativeness and cooperative behavior. This finding suggests that while cooperativeness influences Duchenne smiling with gaze and cooperative behavior independently, nonverbal behavior does not explain the pathway from personality traits to cooperative actions. One explanation for the results is that Duchenne smiles with gaze are not the only emotional expressions displayed by cooperators. As Schug et al. [13] demonstrated, cooperators exhibit both positive and negative emotions. For example, empathetic negative emotions in response to unfair situations may also indicate prosocial preferences [59]. In our study, the participants engaged in ten-minute unstructured conversations that likely included various emotional expressions, not solely positive ones. Furthermore, in this study we employed the classic Ekman and Friesen framework [43] to categorize smiles as Duchenne smiles (genuine) and non-Duchenne (fake) smiles. While this approach is standard, recent studies challenge the notion that smiles can simply be categorized as reflecting true or faked emotions, as smiles can occur in diverse emotional states like distress and pride [60,61,62]. In this study, smiles labeled as Duchenne might have included both positive and negative emotional expressions with varying social functions. Given the significant association between valence and cooperative behavior, further research is necessary to isolate Duchenne smiles that express positive emotions and assess their impact on the relationship between altruism and cooperation.

Furthermore, while the link between the Duchenne smile and cooperation has been explored in various cultural contexts, most research has concentrated on Western cultures [5,6,7,10,13,14,15], leaving a gap in the studies involving Eastern cultures. By investigating the relationship between the Duchenne smile and cooperation within an Eastern cultural framework, this study has made an important contribution to the literature, broadening our understanding of this phenomenon across diverse cultural settings.

IBS has been reported to be associated with prosocial behaviors such as cooperation, coordination, and collective performance [28,29,30]. However, the majority of hyperscanning studies have recorded IBS during structured cooperative interactions [50,63,64,65,66,67]. Some studies have observed IBS while participants perform tasks that also involve elements of naturalistic interaction, such as eye contact and communication with each other [68,69,70,71]. However, under these settings, it is unclear whether the observed IBS was driven by cooperation or by the elements of naturalistic interaction. In comparison, the IBS observed in this study emerged in naturalistic communication during which the participants were not performing any structured tasks or aiming for an instructed collective goal. Our path analysis result showed that cooperativeness significantly predicted Duchenne smiling with gaze and cooperative behavior, however, against previous research in which IBS emerged from structured tasks [65,72,73,74]; IBS during conversation did not predict successive cooperative behavior. A possible explanation for the difference is whether the interactions being used encouraged cooperation. Compared to the naturalistic unstructured conversation used in the current study, previous research used interactions that encouraged participants to cooperate. For example, in a study of IBS during a key-pressing task, task-related IBS was found to be associated with the participants’ mutual inclination to help after the task [72]. In another study of IBS during a naturalistic positive interaction about planning a fun day to spend together, IBS was found to be correlated with self-reported collaboration [73]. However, the authors reported that this effect did not survive FDR correction and called for caution in future research. Our findings revealed the direct effect of cooperativeness on cooperative behavior and a lack of mediation by IBS, indicating that dispositional factors may play a more substantial role than moment-to-moment interpersonal neural synchrony in predicting cooperative outcomes. This suggests that inherent personality traits, such as cooperativeness, are crucial in determining how individuals engage in cooperative interactions, overshadowing the immediate effects of situational dynamics or emotional expressions. Our result that IBS did not predict cooperative behavior also highlights an important aspect of our results. Since IBS in this study spontaneously emerged from naturalistic unstructured communication without a specific task or common goal, it appears that these styles did not inherently drive cooperative behavior. This finding suggests that the contexts in which interpersonal dynamics develop may significantly influence their relevance to cooperative outcomes. Future studies should carefully consider the origins of IBS when designing experimental tasks. For instance, it would be beneficial to investigate how structured contexts, such as collaborative problem-solving or shared goal-oriented tasks, might elicit different interpersonal dynamics compared to unstructured interactions. Understanding the contextual triggers for IBS could provide insight into when and how these styles influence cooperation.

The findings of this study raise important questions about the implications of the lack of mediation by Duchenne smiles and IBS on the relationship between personality traits and cooperative behavior. The absence of mediation suggests that the interplay between emotional expressiveness and prosocial behavior may be more complex than previously understood. We cannot reject the possibility that personality traits, such as cooperativeness, might influence cooperative behavior independently of emotional expressions. It is also possible that alternative mediating factors could clarify the relationship between personality and cooperation. Shared intentionality is one possible mediator, referring to collaborative interactions in which individuals have a mutual goal and coordinated action to pursue [72,75]. Another possible mediator is perceived similarity [76]. Existing studies indicate that perceived similarity among individuals blurs the self-other distinction, thus promoting prosociality [77]. Future studies could benefit from considering potential factors, such as shared intentionality and perceived similarity, that might mediate the relationship between personality and cooperative behavior.

### Limitations and Future Directions

Some cautions are called for regarding the current study. First, like much work on interpersonal synchrony and cooperation, our participants were limited to university and college students. In addition, our participants were East Asians who differ in culture from Western people. Some cross-cultural studies suggest that people’s culture shapes how they judge smiles [78,79]. In order to reach a broader generalization, further research should be conducted on a more diverse and representative population. Studies across diverse cultural groups can shed light on how cultural norms influence interpersonal dynamics and cooperative behavior. Future studies could examine how collectivist versus individualist cultures shape the judgment of smiles and their effects on cooperation. Moreover, in the current study, we focused on the left prefrontal region using two-channel fNIRS. Recent fNIRS hyperscanning studies suggest that the middle frontal cortex and right dorsolateral prefrontal cortex also play crucial roles in interpersonal synchrony and its prosocial consequences [72,80,81]. In this study, two-channel fNIRS devices were used due to practical considerations, including their portability and ease of use in naturalistic settings. However, this may have limited the spatial resolution of the findings. Future studies regarding brain activity during interpersonal synchrony should take into account these regions by employing a multi-channel fNIRS hyperscanning approach. The absence of the expected mediating effects from Duchenne smiling with gaze in our study prompts a reevaluation of the factors influencing cooperativeness. In this study, our focus was limited to nonverbal cues such as Duchenne smiles and gazes. While these facial expressions are often highlighted in research on interpersonal communication [82], other nonverbal signals, such as body language, posture, and gestures, may also play crucial roles in facilitating cooperation. Future research should explore how these additional nonverbal cues interact with cooperative behaviors, potentially offering a more comprehensive understanding of the dynamics at play. Our study utilized an unstructured communication task for interaction, which may not have adequately captured the nuances of cooperative communication. When interactions are structured under a cooperative framework, the nature of communication may change significantly. Future studies incorporating a context where communication is explicitly aimed at achieving cooperative outcomes, such as in team-based tasks or collaborative projects, could yield different insights into the role of IBS.

One limitation of this study is that while the statistical power for the mediation analysis is adequate (0.81), it could be improved for path analysis (0.63). To enhance statistical power in future studies, it would be beneficial to either increase the number of participants or adjust the research design accordingly.

## 5. Conclusions

The current study investigated the causal relationship between cooperativeness, Duchenne smiling with gaze, IBS, and cooperative behavior. The findings from both the mediation and path analyses consistently underscored that cooperative behavior is directly influenced by the personality trait of cooperativeness. Despite observing significant associations between cooperativeness and Duchenne smiling with gaze, neither Duchenne smiling with gaze nor IBS mediated the relationship between cooperativeness and cooperative behavior. These results highlight the pivotal role of individual dispositions of cooperativeness in shaping prosocial behaviors, shedding light on the nuanced mechanisms underlying cooperative interactions in social settings. Moreover, this study, set in an Eastern cultural context, broadens our understanding of the relationship between the Duchenne smile and cooperation by adding cultural diversity to the existing research. The findings of this study suggest that while cues such as Duchenne smiles and gazes can serve as indicators of cooperativeness, they do not function as mediators in cooperative behavior. This distinction is crucial, as it implies that simply enhancing nonverbal communication skills may not be sufficient for fostering cooperative relationships. In the realm of education, this suggests a shift in focus from merely teaching nonverbal communication techniques to fostering an environment that encourages the development of cooperative traits. Recent research also pointed out the importance of considering the relationship between personality traits with interaction and engagement to increase students’ academic performance through collaborative works [16]. In summary, while nonverbal cues are valuable indicators of cooperativeness, the enhancement of intrinsic cooperative traits through educational practices is essential for building effective collaborative relationships. Future research could further explore the additional factors that may modulate these relationships, enhancing our understanding of the complex interplay between personality traits and cooperative behaviors.

## Figures and Tables

**Table 1:** The incentive structure of the prisoner’s dilemma game used in the current study expressed as a payoff matrix.

Participant A’s Cooperation Level (i.e., How Much A Gives)					
Participant B’s cooperation level		150	100	…	0
	150	300R300	200R350		0R450
	100	350R200	250R250		50R350
	…				
	0	450R0	350R50		150R150

**Table 2:** The mediating effect of Duchenne smiling with gaze in the relationship between cooperativeness and cooperative behavior.

	Effect	95% CI Lower	95% CI Upper	*p*-Value
Total effect	0.661	0.415	0.91	<2 × 10^−16^
Direct effect	0.599	0.339	0.86	<2 × 10^−16^
Indirect effect	0.062	−0.056	0.20	0.25
Proportion mediated	0.085	−0.077	0.30	0.24

**Table 3:** Results of two-sample *t*-test of coherence from genuine dyads and permutated dyads between timescale 15.48 and 21.89 s (0.04–0.06 Hz).

Period	Estimate (Genuine)	Estimate (Permutated)	*t*-Value	Df	*p*-Value	FDR-Adjusted Q-Value	Cohen’s d
15.48	0.336	0.312	2.726	65.955	0.0081	0.0479	0.399
16.40	0.344	0.309	3.993	64.634	0.0001	0.0016	0.592
17.37	0.352	0.305	4.896	59.746	<8 × 10^−6^	0.0001	0.765
18.41	0.357	0.304	5.222	55.929	<3 × 10^−6^	0.0001	0.859
19.50	0.359	0.306	4.842	53.705	<2 × 10^−5^	0.0001	0.825
20.66	0.359	0.311	4.006	51.616	0.0001	0.0016	0.708
21.89	0.358	0.316	3.103	48.912	0.0031	0.0217	0.580

## Data Availability

Data are contained within the article or Supplementary Materials. The original contributions presented in the study are included in the article and the Supplementary Materials, further inquiries can be directed to the corresponding author.

## Supplementary Materials

The following supporting information can be downloaded at https://www.mdpi.com/article/10.3390/bs14110987/s1, Minimal dataset.
